# Up-Regulation of Urotensin II and Its Receptor Contributes to Human Hepatocellular Carcinoma Growth via Activation of the PKC, ERK1/2, and p38 MAPK Signaling Pathways

**DOI:** 10.3390/molecules191220768

**Published:** 2014-12-12

**Authors:** Xiao-Tong Yu, Peng-Yan Wang, Zheng-Ming Shi, Kun Dong, Ping Feng, Hong-Xia Wang, Xue-Jiang Wang

**Affiliations:** 1 Department of Physiology and Pathophysiology, School of Basic Medical Sciences, Capital Medical University, No.10 Xitoutiao, You An Men, Beijing 100069, China; E-Mails: yxt20053@sina.com (X.-T.Y.); ping-feng1002@163.com (P.F.); 2 Department of Pathology, Peking Union Medical Hospital, Beijing 100692, China; E-Mail: wpy1120@163.com; 3 Department of General Surgery, Beijing Jishuitan Hospital, Beijing 100031, China; E-Mail: shi-zm@163.com; 4 Department of Pathology, Beijing Youan Hospital, Capital Medical University, Beijing 100069, China; E-Mail: bugu_bird_logo@163.com

**Keywords:** urotensin II, human hepatocellular carcinoma, cell proliferation, signaling pathway

## Abstract

Urotensin II (UII) and its receptor (UTR) have mitogenic effects on tumor growth. Our previous study demonstrated that the UII/UTR system is up-regulated in dithyinitrosamine-induced precancerous rat liver lesions. However, its role in human hepatocellular carcinoma remains unknown. In this study, the mRNA and protein expression of UII and its receptor (UTR) in human hepatocellular carcinoma samples and in the BEL-7402 human hepatoma cell line were evaluated. In addition, the effect of exogenous UII on the pathways that regulate proliferation in BEL-7402 cells *in vitro* were determined. Liver sections were subjected to immunohistochemical staining. mRNA expression was detected by real-time polymerase chain reaction analysis, and protein levels were evaluated by western blotting. Proliferating cells were detected by BrdU incorporation. The expression of UII/UT mRNA and protein significantly increased in human hepatocellular carcinoma samples, and in BEL-7402 cells. Administration with UII increased the phosphorylation of protein kinase C (PKC), extracellular signal-regulated kinase (ERK1/2) and p38 mitogen-activated protein kinases (p38 MAPK). Furthermore, GF109203x, PD184352, and SB203580 partially abolished UII-induced proliferation of BEL-7402 cells. These results provide the first evidence that up-regulation of the UII/UT system may enhance proliferation of the human hepatoma cell line at least in part via PKC, ERK1/2, and p38 MAPK signaling pathways, and may provide novel therapeutic targets for inhibiting human hepatocellular carcinoma.

## 1. Introduction

Urotensin II (UII), a somatostatin-like cyclic peptide, was first isolated from the urophysis of teleost fishes, and later from mammals and humans [1,2]. Human UII is an undecapeptide that retains the cyclic portion typical of fish UII. UII has been shown to recognize the orphan G-protein coupled receptor (GPCR) GPR14 [3]. Subsequently, a human GPCR displaying high sequence similarity to rat GPR14 was cloned and renamed urotensin-II (UT) receptor [4]. UII and UT have been detected in various human tissues, including the liver [5]. Originally, UII was detected as a potent vasoconstrictor in mammalians, but it has since been reported to stimulate calcium mobilization in cells stably transfected with its receptor [6]. Because calcium is an important activator of signal transduction pathways involved in cell proliferation through protein kinase C (PKC)-dependent signaling. UII is thought to act on tumor cells, affecting cell growth as an autocrine/paracrine factor [6,7,8].

UII has been reported to be involved in the pathogenesis of some tumors. Takashashi *et al.* [8] showed that UII and UT are expressed in many tumor cell lines, such as the T98G glioblastoma cell line, the IMR32 neuroblastoma cell line, the BeWo choriocarcinoma cell line, the SW-13 adrenocortical carcinoma cell line, the DLD-1 colorectal carcinoma cell line, and the HeLa cervical carcinoma cell line. Furthermore, UII significantly stimulates the proliferation of some tumor cells, including SW-13 cells [9], VMRC-RCW renal cell carcinoma cells [9], and human pheochromocytoma cells [10]. Recently, Federico *et al.* showed UT is over-expressed in colon cancer cell lines and in colon carcinoma in humans, and UT-related pathway may play a role in colon carcinogenesis [11]. Our previous study demonstrated that the UII/UT system is up-regulated in dithyinitrosamine-induced rat precancerous liver lesions [12]; however, no report has been published on the expression of this system in human hepatocellular carcinoma.

In this study, using a combined* in vivo* and *in vitro* approach, the expression of the UII/UT system in human hepatocellular carcinoma and in the BEL-7402 human hepatocellular cell line were investigated, and the effect of exogenous UII on the phosphorylation of PKC, ERK1/2, and p38 MAPK was examined to determine the possible mechanism underlying the pathogenesis of hepatocellular carcinoma.

## 2. Results and Discussion

### 2.1. Hematoxylin and Eosing Staining and Alpha-Fetoprotein Expression in the Liver

**Figure 1:**
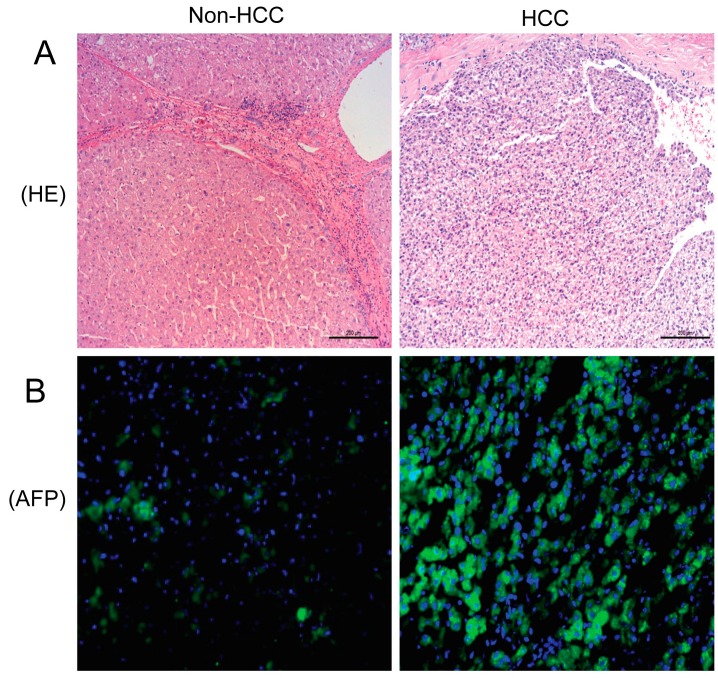
H&E staining (**A**) and immunofluorescence staining of AFP in non-HCC and HCC tissues (**B**). The green stained areas are AFP-positive foci.

Hematoxylin and eosin (H&E) staining of liver tissues showed nodular hepatocellular carcinoma (HCC). Immunofluorescence analysis revealed almost no α-fetoprotein (AFP) expression in adjacent noncancerous liver tissues, but its expression significantly increased in HCC specimens (Figure 1).

### 2.2. mRNA and Protein Expression of UII and UT in HCC

**Figure 2:**
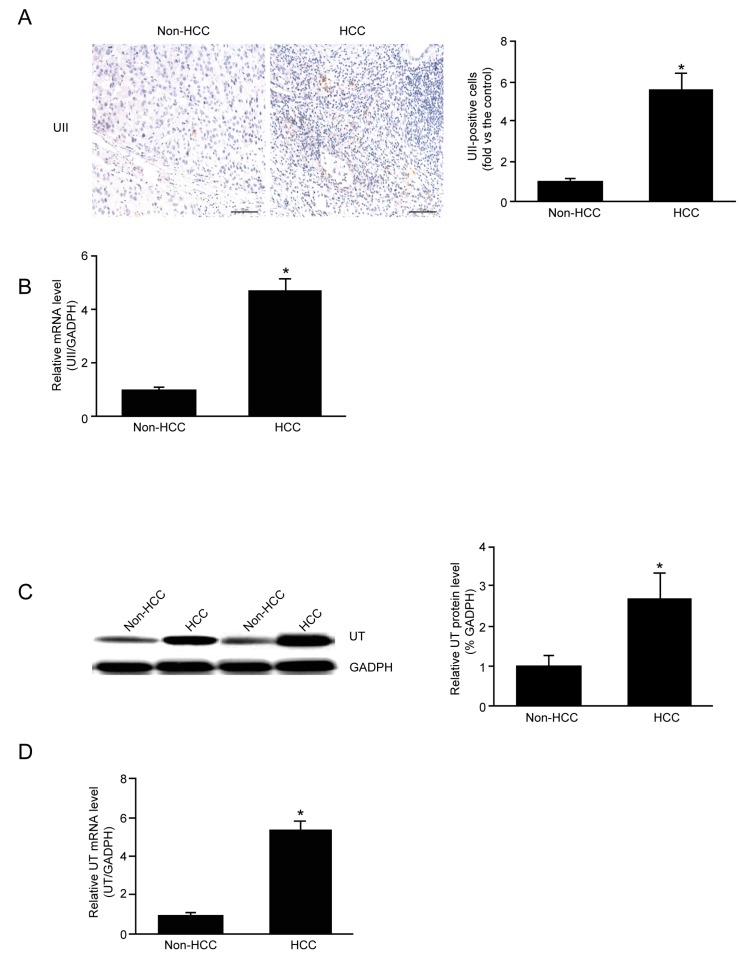
UII/UT mRNA and protein expression in non-HCC and HCC tissues. (**A**) Analysis of UII protein expression by immunohistochemical staining; (**B**) Quantitative analysis of UII mRNA expression by qPCR; (**C**) Analysis of UT protein expression by western blotting; (**D**) Quantitative analysis of UT mRNA expression by qPCR. Data are mean ± SE (*n* = 8, * *p* < 0.05 compared with non-HCC).

To investigate the role of the UII/UT system in HCC, the expression of endogenous UII and UT in liver tissue was first examined. Quantitative real-time PCR (qPCR) analysis revealed that the mRNA expression of UII and UT was significantly higher in HCC compared to non-HCC tissue (Figure 2B,D). Immunohistochemical staining revealed the increased protein expression of UII in HCC (Figure 2A), and western blot analysis showed higher UT protein expression in HCC compared to non-HCC tissue (Figure 2C).

### 2.3. The Phosphorylation of PKC, ERK, and p38 MAPK Was Increased in HCC

**Figure 3:**
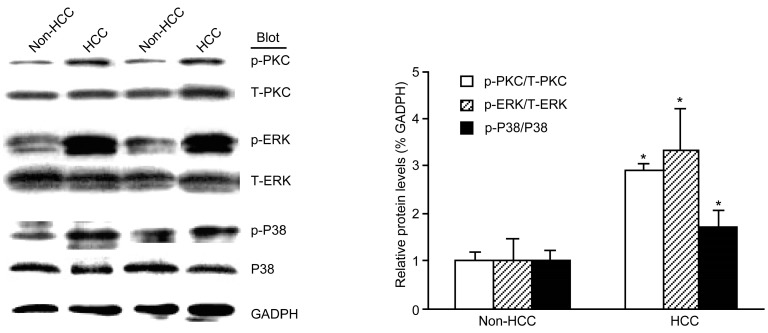
HCC induced the phosphorylation of PKC, ERK, and p38 MAPK. Western blot analysis and quantitation of PKC, ERK, and p38 MAPK normalized against GADPH. Data are mean ± SE (*n* = 5, * *p* < 0.05 compared with non-HCC).

Western blot analysis revealed that the phosphorylation of PKC, ERK, and p38 MAPK significantly increased in HCC compared with non-HCC tissue, and the phosphorylation of PI3K was not increased in HCC compared with non-HCC tissue (data not shown) (Figure 3).

### 2.4. Up-Regulation of the UII/UT System in BEL-7402 Cells

**Figure 4:**
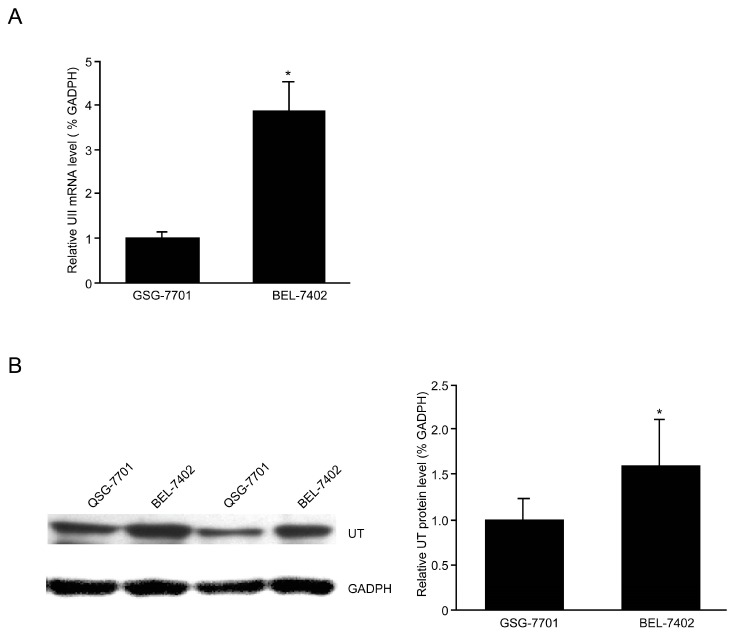
UII/UT system was up-regulated in BEL-7402 cells. (**A**) Expression of UII mRNA by qPCR; (**B**) Expression of UT protein by western blot analysis. Data are mean ± SE (* *p* < 0.05 compared with QSG-7701).

BEL-7402 is a HCC cell line that is extensively used in the field of hepatoma research in China, whereas QSG-7701 is a cell line derived from the peripheral tissue of liver carcinoma. BEL-7402 and QSG-7701 cell lines were used to detect the expression of the UII/UT system. qPCR analysis revealed that the UII mRNA expression level was significantly higher in BEL-7402 than QSG-7701 cells, and qPCR and Western blotting showed that UT mRNA and protein expression levels, respectively, were significantly higher in BEL-7402 compared to QSG-7701 (Figure 4).

### 2.5. UII Induced the Phosphorylation of PKC, ERK, and p38 MAPK in BEL-7402 Cells

**Figure 5:**
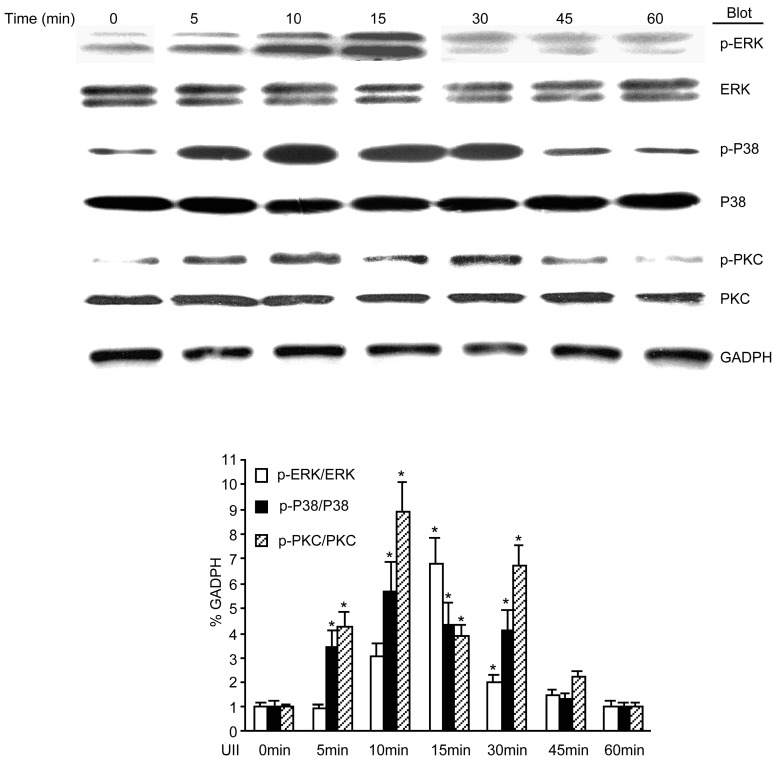
UII induced the phosphorylation of PKC, ERK, and p38 MAPK in BEL-7402 cells. BEL-7402 cells were treated with 100 nmol/L UII for various periods of time, and the phosphorylation of PKC, ERK, and p38 MAPK were detected by western blot analysis. Data are mean ± SE (*n* = 5, * *p* < 0.05 compared with QSG-7701).

Because the phosphorylation of PKC, ERK, and p38 MAPK increased in HCC tissues, it was further investigated whether the exogenous administration of UII could induce phosphorylation in BEL-7402 cells. The results showed that in cells treated with 10^−7^ M UII, the phosphorylation of PKC, ERK, and p38 MAPK increased at 5 min, peaked at 10 min, and was sustained for up to 45 min compared to untreated cells (Figure 5).

### 2.6. UII Increased the Proliferation of BEL-7402 Cells via Phosphorylation of PKC, ERK, and p38 MAPK

**Figure 6:**
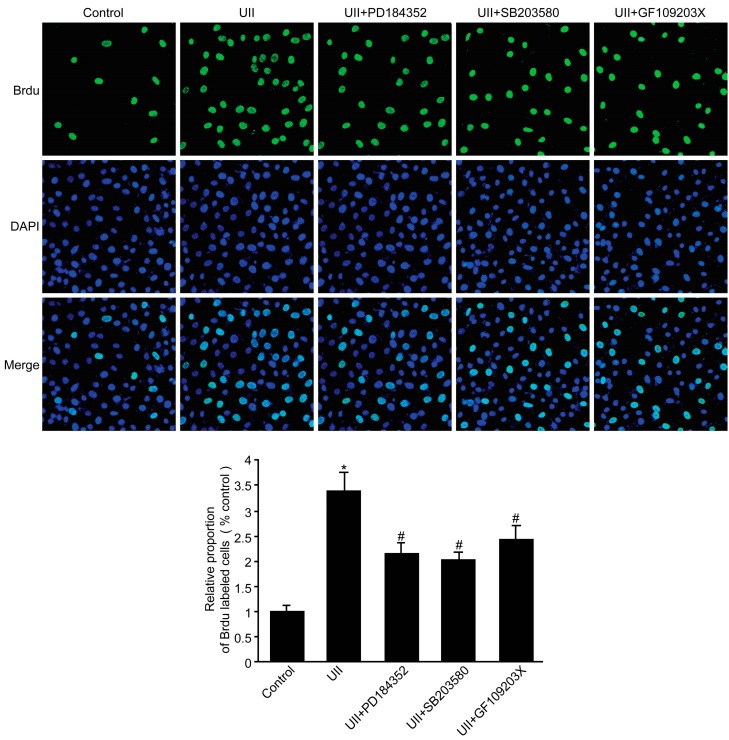
UII increased BEL-7402 cell proliferation via the phosphorylation of PKC, ERK, and p38 MAPK. BEL-7402 cells were pretreated with GF109203x (100 nmol/L), PD184352 (1 mmol/L) or SB203580 (10 mmol/L) 30 min before a 24-hour incubation with 100 nmol/L UII. The proliferation of BEL-7402 cells was determined with the bromodeoxyuridine (BrdU) incorporation assay. Data are mean ± SE (*n* = 6, * *p* < 0.05 compared with the control; # *p* < 0.05 compared with UII).

To examine whether phosphorylation of PKC, ERK, and p38 MAPK is required for the UII-induced proliferation of BEL-7402 cells, the cells were pretreated with PKC inhibitor GF109203x, the ERK inhibitor PD184352, and the p38 MAPK inhibitor SB203580. As shown in Figure 6, treatment with UII significantly increased cell proliferation compared to control cells, and treatment with GF109203x, PD184352, and SB203580 inhibitors partially abolished UII-induced BEL-7402 cell proliferation via inhibition of PKC, ERK, and p38 MAPK respectively, but the inhibitor effects observed after UII treatment could be due to UII-mobilized pathways.

### 2.7. Discussion

In the present study, it was demonstrated that the expression of the UII/UT system increased in HCC tissue and cell lines, and that the exogenous administration of UII increased the expression of transcription factors and induced the activation of signaling pathways that regulate the proliferation of cancer cells, such as PKC, ERK, and p38 MAPK. Moreover, GF109203x, PD184352, and SB203580 inhibitors partially abolished UII-induced BEL-7402 cell proliferation. To the best of our knowledge, these results provide the first evidence of UII/UT up-regulation in HCC, caused, at least in part, via activation of PKC, ERK1/2, and p38 MAPK signaling pathways.

HCC accounts for >90% of all primary liver cancers, is the fifth most prevalent cancer in the world, and is the third cause of global liver cancer mortality, with a 5% survival rate over 5 years and more than 500,000 deaths annually [13]. The development of HCC is generally thought to be due to alcohol abuse, persistent infection with hepatitis virus B and C, or other metabolic disorders that lead to liver cirrhosis. However, the mechanism underlying HCC remains unclear. Some vasoactive peptides are known to be produced and secreted by tumor cells, and act as paracrine growth stimulators [14]. Consistent with our previous results, the present study shows that expression of the UII/UT system was higher in HCC cells compared to non-HCC cells, and was also higher in BEL-7402 cells compared to QSG-7701 cells (Figure 1 and Figure 4). However, the mechanism by which the up-regulation of the UII/UT system is involved in HCC remains unclear.

UII is recognized by its receptor, UT, resulting in generation of second messengers [15]. These second messengers can trigger Ca^2+^ release [16], subsequently activating Ca^2+^-dependent protein kinases, such as PI3K, PKC, ERK, and p38 MAPK [15,17]. Some studies have shown that PKC and ERK phosphorylation are required for proliferation of various cell types and cell lines involved in many diseases [18,19]. Our previous studies demonstrated that the phosphorylation of PKC and ERK1/2, but not p38 MAPK, was activated by UII in rat precancerous liver lesions [12]. Consistent with this, the present study showed that phosphorylation of PKC and ERK1/2 were significantly increased in HCC tissues and BEL-7402 cells. Inconsistent with this, the phosphorylation of p38 MAPK was also increased in HCC tissues maybe resulting from different species. The phosphorylation of PI3K was not increased in HCC (data not shown). Here, the pattern of UII-induced signaling observed in HCC and involving PKC, ERK1/2, and p38 MAPK, but not PI3K, is consistent with the set of cellular events triggered by UII in cardiomyocytes [20], but exhibits some difference with UT-transfected cell lines involving ERK1/2 and PI3K, but not p38MAPK [21]. More importantly, inhibitors of PKC, ERK1/2, and p38 MAPK partially abolished the UII-induced proliferation of BEL-7402 cells. Collectively, our results support the fact that UII can induce the proliferation of BEL-7402 cells via PKC/ERK/p38 MAPK signaling pathways.

## 3. Experimental Section

### 3.1. Patients

The study protocol was approved by the ethics committee of the Capital Medical University, and informed consent was given by each patient before enrollment. The consent was verbal because the samples were the residues of clinical test specimens. Thus, no additional treatment of the patients was required.

Human hepatocellular carcinoma samples were obtained from Beijing YouAn Hospital, Capital Medical University. Exclusion criteria included type 2 diabetes mellitus, cardiovascular diseases, and hypertension. Samples were obtained from a series of consecutive, unselected patients who underwent radical resection of HCC. HCC samples and non-HCC samples from the same patients were used to measure UII/UT protein expression and phosphorlation of PKC, ERK1/2, p38 MAPK and PI3K.

### 3.2. Materials

TRIzol™ reagent and a reverse transcription system were purchased from Promega (Madison, WI, USA). Sequences of oligonucleotide primers for RT-PCR analysis were synthesized by Sai Bai Sheng (Beijing, China). PrimeScript™ RT master mix and PrimeScript™ RT reagent kit with gDNA Eraser were purchased from TaKaRa (Otsu Shiga, Japan). The western blot kit and anti-UT and anti-glyceraldehyde-3-phosphate dehydrogenase (GADPH) antibodies were purchased from Santa Cruz Biotechnology (Santa Cruz, CA, USA). Antibodies directed against p38 mitogen-activated protein kinase (MAPK), phosphorylated p-P38, extracellular signal-regulated protein kinases 1 and 2 (ERK1/2), p-ERK1/2, protein kinase C (PKC), and p-PKC were purchased from Cell Signaling Technology (Danvers, MA, USA). The p38 MAPK inhibitor SB203580, PKC inhibitor GF109203x, and ERK1/2 inhibitor PD184352 were purchased from Sigma-Aldrich (St Louis, MO, USA). All chemicals were of analytical grade.

### 3.3. Cell Culture and Treatment

The QSG-7701 human liver cell line and BEL-7402 human hepatoma cell line were purchased from the Center Cell Resources of the Shanghai Institute of Life Science affiliated with the Chinese Academy of Sciences. The cell lines were cultured in RPMI1640 medium supplemented with 10% fetal bovine serum and antibiotics (100 U/mL penicillin and 100 μg/mL streptomycin). In some experiments, cultured BEL-7402 cells were pretreated for 30 min with SB203580 (10 mmol/L), PD184352 (1 mmol/L), or GF109203x (100 nmol/L) to inhibit p38 MAPK, ERK1/2, and PKC, respectively.

### 3.4. Quantitative Real-Time PCR Analysis

Total RNA was extracted from cultured cells or fresh liver tissue using the TRIzol^®^ method. RNA samples (1 µg) were reverse-transcribed to generate first-strand complementary DNA (cDNA). Quantitative real-time PCR (qPCR) analysis was performed as previously described [22]. The primers for UII, UT, and GADPH were designed by Sangon Biotech (Shanghai, China).

### 3.5. Western Blot Analysis

Western blot analysis was performed as previously described [23]. The cells and liver tissues were lysed with lysis buffer (20 mM Tris, pH 7.5; 1 mM EDTA; 150 mM NaCl; 1 mM EGTA; 1 mM β-glycerophosphate; 1% Triton™ X-100; 2.5 mM sodium pyrophosphate; 1 mM Na3VO4; 4 mg/mL aprotinin; 4 mg/mL leupeptin; 4 mg/mL pepstatin; Ang II+ and 1 mM phenylmethanesulfonyl fluoride [PMSF]). After clarification by centrifugation, 50–60 ug protein lysate was separated on 10% SDS–PAGE gels and then transferred to polyvinylidenedifluoride (PVDF) membranes. The membranes were incubated with primary antibodies against UT, p-ERK1/2, ERK1/2, p-p38 MAPK, p38 MAPK, p-PKC, and PKC (all at a dilution of 1:800–1000), as well as GADPH (1:5000 dilution). Membranes were washed and incubated with horseradish peroxidase (HRP)-conjugated secondary antibodies (1:1,000 dilution). Signals from the phosphorylated protein forms were normalized to the amount of total target protein and GADPH.

### 3.6. Immunohistochemistry Assay

Immunohistochemistry assay was performed as previously described [12]. Liver tissue was fixed in 4% paraformaldehyde solution, embedded in paraffin, and sectioned at a thickness of 4–5 µm. Endogenous peroxidase activity was quenched by incubation in 3% hydrogen peroxide (H_2_O_2_) for 15 min. The sections were then blocked with 5% bovine serum albumin (BSA) to prevent nonspecific staining from the secondary antibody. After incubation overnight at 4 °C with primary antibody, the sections were washed with phosphate-buffered saline (PBS) and incubated with HRP-conjugated secondary antibody at room temperature (RT) for 1 h, and then washed in PBS and incubated in diaminobenzidine. Positive cells were visualized by light microscopy (20×, Leica, Wetzlar, Germany). Omission of primary antibody and staining with a non-immune IgG (isotype control) served as negative controls.

### 3.7. BrdU Incorporation

After 12 h of serum deprivation, BEL-7402 cells were treated with 10^−7 M UII for 24 h. ^Bromodeoxyuridine (BrdU; final concentration, 10 mmol/L) was added to the media for the final 1 h. Cells were washed with PBS and fixed with 4% paraformaldehyde for 30 min. The cells were incubated with monoclonal anti-BrdU antibody followed by secondary antibody. After being washed, the nuclei were counterstained with 4,6-diamidino-2-phenylindole (DAPI) for 5 min. The slides were visualized with an fluorescence microscope (20×, Olympus, Tokyo, Japan).

### 3.8. Statistical Analysis

Data are expressed as mean ± SEM. Statistical differences between two groups were compared using a two-tailed unpaired Student’s *t*-test. Differences between multigroups were analyzed by one-way analysis of variance (ANOVA), followed by a Student-Newman-Keuls test. A *p* value < 0.05 was considered statistically significant. All statistical analyses were performed with Statistical Package for the Social Sciences (SPSS) version 12.0 software (SPSS Inc., Chicago, IL, USA).

## 4. Conclusions

Our results provide novel evidence for the important role of the UII/UT system in HCC. The up-regulation of the UII/UT system in HCC increases BEL-7402 cell proliferation, in part, via activation of PKC, ERK1/2, and p38 MAPK signaling pathways. Collectively, these findings confirm the engagement of UII/UT system-mediated cell proliferation in HCC, and shed light on possible new strategies for the prevention and control of HCC.
